# Acupuncture attenuates hypertension-induced myocardial hypertrophy: role of the SIRT1/NF-κ B/MAPK signaling axis in the hypothalamic paraventricular nucleus

**DOI:** 10.3389/fnins.2026.1982426

**Published:** 2026-10-14

**Authors:** Jing Zhang, Jiali Wu, Peng Wang, Binghui Wang, Songli Li, Guirong Luo, Zichun Xiao, Qingguo Liu

**Affiliations:** School of Acupuncture-Moxibustion and Tuina, Beijing University of Chinese Medicine, Beijing, China

**Keywords:** acupuncture, hypertension, hypothalamic paraventricular nucleus, myocardial hypertrophy, neuroinflammation, SIRT1/NF-κB/MAPK signaling axis

## Abstract

**Background:**

Chronic hypertension is a major risk factor for cardiovascular complications and can induce pathological myocardial hypertrophy. The hypothalamic paraventricular nucleus (PVN) is a key autonomic regulatory center involved in hypertension through sympathetic overactivation, neuroinflammation, and central renin–angiotensin system dysregulation. Although acupuncture exerts protective effects against hypertension-related target organ damage, its central mechanisms remain unclear. This study investigated whether acupuncture was associated with the attenuation of hypertension-induced myocardial hypertrophy in spontaneously hypertensive rats (SHRs) and whether these effects may involve modulation of the sirtuin 1 (SIRT1)/nuclear factor-κB (NF-κB)/mitogen-activated protein kinase (MAPK) signaling axis in the PVN.

**Methods:**

Twenty-seven SHRs were randomly assigned to Model, Acupuncture, and Losartan groups (*n* = 9 per group), while nine Wistar-Kyoto (WKY) rats served as Controls. Rats received acupuncture at Taichong (LR3), Baihui (GV20), and Sanyinjiao (SP6) or losartan treatment for 12 weeks. Blood pressure, plasma norepinephrine (NE), cardiac function, myocardial remodeling, fibrosis, and PVN-related molecular changes were assessed.

**Results:**

Acupuncture reduced systolic and diastolic blood pressure and decreased plasma NE levels in SHRs. In the PVN, acupuncture increased SIRT1 expression and inhibited NF-κB p65 expression and MAPK phosphorylation. Moreover, acupuncture suppressed central renin–angiotensin system activation, neuroinflammation, and neurotransmitter imbalance by regulating ACE, AT1R, pro-inflammatory cytokines, GABA, and Glu levels. Echocardiographic and histological analyses demonstrated that acupuncture alleviated myocardial hypertrophy, improved cardiac function, and reduced fibrosis and structural remodeling.

**Conclusion:**

Acupuncture was associated with the attenuation of hypertension-induced myocardial hypertrophy in SHRs, possibly involving modulation of the PVN SIRT1/NF-κB/MAPK signaling axis. These effects may involve suppression of neuroinflammation and sympathetic overactivation, regulation of the central renin–angiotensin system, and restoration of PVN neurotransmitter balance, providing new insights into the central neural mechanisms underlying acupuncture-mediated cardioprotection in hypertension.

## 1 Introduction

Hypertension remains one of the leading modifiable determinants contributing to the global burden of cardiovascular diseases and related mortality (Xu et al., 2026). Persistent elevation of blood pressure (BP) induces vascular structural remodeling and functional impairment, while simultaneously promoting maladaptive myocardial growth and ventricular remodeling, thereby increasing the risk of adverse cardiovascular outcomes, including heart failure (Nemtsova et al., 2023; Mutai et al., 2026). Despite the availability of various antihypertensive therapies, effective prevention of hypertension-associated target organ damage remains challenging, highlighting the necessity of exploring adjunctive and alternative therapeutic strategies.

Accumulating evidence indicates that the hypothalamic paraventricular nucleus (PVN) serves as a critical central integrative hub linking neural regulation with cardiovascular homeostasis through modulation of autonomic activity and sympathetic outflow (Savić et al., 2022; Gao et al., 2024; Sun et al., 2026). The PVN maintains cardiovascular homeostasis primarily by modulating sympathetic outflow (Dampney et al., 2018; Duque et al., 2026). Recent studies have demonstrated that PVN dysfunction, characterized by activation of the local renin–angiotensin system (RAS), neuroinflammation, and imbalance between excitatory and inhibitory neurotransmission, contributes to sympathetic excitation and hypertensive cardiovascular remodeling (Jia et al., 2023).

As a key NAD^+^-dependent deacetylase, sirtuin 1 (SIRT1) preserves cellular homeostasis by modulating gene transcription and regulating inflammatory, apoptotic, and oxidative stress-related processes (Liu et al., 2022; Yang et al., 2022; Chang et al., 2026). Within the cardiovascular system, SIRT1 activation protects against ischemia-reperfusion damage, restrains cardiac hypertrophy, and improves endothelial function (Chen et al., 2021). Importantly, SIRT1 exerts a negative regulatory effect on nuclear factor-κB (NF-κB)-mediated transcription, while suppressing mitogen-activated protein kinase (MAPK) signaling pathways, including c-Jun N-terminal kinase (JNK), p38-MAPK, and extracellular signal-regulated kinase 1/2 (ERK1/2) (Song et al., 2022; He et al., 2023; Jia et al., 2023). Therefore, dysregulation of the SIRT1/NF-κB/MAPK axis within the PVN may represent a potential molecular link between neuroinflammation, sympathetic overactivation, and hypertensive cardiac remodeling.

Acupuncture, a widely used non-pharmacological intervention derived from traditional Chinese medicine, has demonstrated antihypertensive effects, target organ protection, and improvement of hypertension-related symptoms (Chang et al., 2023; Zhou et al., 2026). Accumulating evidence indicates that these effects may involve the modulation of autonomic activity and neuroinflammatory responses (Li et al., 2022). In spontaneously hypertensive rats (SHRs), our previous studies further demonstrated that acupuncture at LR3 (Taichong), GV20 (Baihui), and SP6 (Sanyinjiao) significantly reduced BP and attenuated myocardial hypertrophy (Ji et al., 2019; Wu et al., 2024). The selection of these three acupoints is also supported by previous clinical and experimental evidence. Stimulation of LR3 has been reported to reduce BP in patients with hypertension and in SHRs, possibly involving central neural mechanisms (Li et al., 2024). Acupuncture at GV20 has also been reported to improve BP, morning BP surge, and BP variability (Zhou et al., 2022), whereas SP6 is frequently included in acupuncture protocols for hypertension, with evidence suggesting its potential involvement in vascular endothelial and inflammatory regulation (Fu et al., 2023; Lin et al., 2023). Nevertheless, the central mechanisms underlying the cardioprotective effects of acupuncture against hypertension-induced myocardial hypertrophy remain poorly understood.

Therefore, the present study investigated whether acupuncture may attenuate myocardial hypertrophy in SHRs and whether modulation of the SIRT1/NF-κB/MAPK axis within the PVN may be involved in these effects. Furthermore, we examined whether these effects may involve changes in RAS activity, inflammatory responses, and neurotransmitter homeostasis, along with alterations in sympathetic activity and cardiovascular remodeling. This study provides new insights into the central neural mechanisms potentially involved in acupuncture-related cardioprotection and expands our understanding of brain–heart interactions in hypertension-related cardiac remodeling.

## 2 Materials and methods

### 2.1 Animals and groups

Twenty-seven SHRs and nine Wistar-Kyoto (WKY) rats (male, 11 weeks of age, body weight 230 ± 30 g) were supplied by Beijing Vital River Laboratory Animal Technology Co., Ltd. (License No. SYXK [Beijing] 2023-0011). All animals were housed under standardized conditions. All experimental procedures were approved by the Institutional Animal Care and Use Committee of Beijing University of Chinese Medicine (Approval No. BUCM-2025040103-2003).

Following a 7-day acclimatization period, the 9 WKY rats were assigned to the Control group. According to a computer-generated randomization sequence, the 27 SHRs were randomly assigned to the Model, Acupuncture, and Losartan groups (*n* = 9 per group).

### 2.2 Interventions

The interventions were administered in the morning, 5 times weekly with a 2-day rest period, for 12 consecutive weeks. All rats were conscious and unanesthetized during the interventions, and all procedures were performed by experienced investigators using a standardized handling and restraint protocol. For the Acupuncture group, rats received needling at GV20, LR3, and SP6; detailed acupoint locations and manipulation procedures are shown in Figure 1. Sterile disposable stainless-steel needles (0.25 × 13 mm; Beijing Zhongyan Taihe Medical Instrument Co., Ltd., Beijing, China) were inserted to a depth of 1–2 mm. GV20 and bilateral LR3 received reducing twirling manipulation for 1 min, whereas bilateral SP6 received gentle twirling manipulation for 1 min, as shown in Figure 1. Meanwhile, rats assigned to the Model and Control groups received no intervention but were subjected to the same restraint procedure for 20 min to ensure equivalent handling conditions. The restraint duration and general handling procedures were kept consistent across groups to minimize differences in stress exposure attributable to the experimental procedures. As a positive control, the Losartan group received once-daily intragastric administration of losartan potassium at 20 mg/kg/day, with the drug solution prepared in distilled water, for 12 weeks.

**FIGURE 1:**
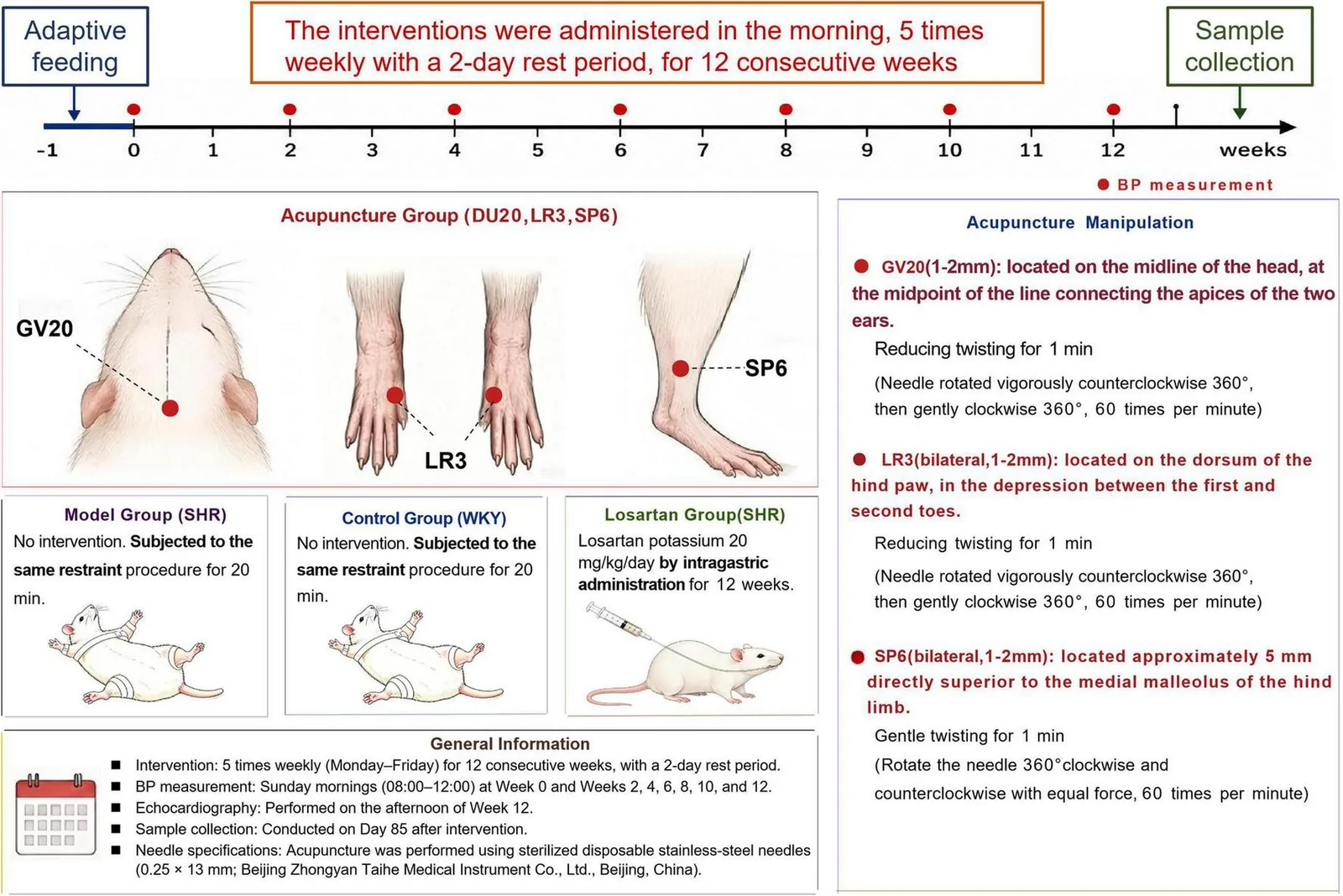
Overview of the rat experimental design, including timeline, grouping, acupoint locations and acupuncture manipulation procedures.

### 2.3 BP measurement

A noninvasive tail-cuff system (BP-2010A; Softron Biotechnology, Beijing, China) was used to determine SBP and DBP under controlled room temperature (20–26 °C). All measurements were performed by two experienced investigators. Before each measurement, rats were placed in restrainers and acclimated on a warming platform for 10 min to promote tail blood flow. Care was taken to minimize handling stress and avoid disturbing the animals during the procedure to reduce BP fluctuations. On Sunday mornings from 08:00 to 12:00, BP was measured at baseline (the day preceding treatment initiation, Week 0) and then at Weeks 2, 4, 6, 8, 10, and 12 throughout the intervention period. At each assessment, three consecutive measurements were recorded per animal, with the mean value entered into the statistical analysis.

### 2.4 Echocardiographic analysis

Rats were maintained under continuous inhalational anesthesia with isoflurane, and the chest hair was cleared with a hair removal cream. A VEVO 2100 system (VisualSonics, Toronto, Canada) was then employed to perform transthoracic echocardiography in accordance with previously described methods (Sahn et al., 1978). Left ventricular posterior wall thickness in diastole (LVPWd), interventricular septal thickness in diastole (IVSd), left ventricular internal diameter in diastole (LVIDd), and left ventricular ejection fraction (LVEF) were measured. For each parameter, the average value derived from three consecutive cardiac cycles was used for subsequent analyses.

### 2.5 Tissue sample collection

On day 85, following completion of all interventions, rats were anesthetized to a deep state with sodium pentobarbital (30 mg/kg, intraperitoneally) and euthanized by controlled blood withdrawal. Blood samples, brain tissues, and hearts were then collected. As described previously (Palkovits, 1973; Coppola et al., 2004), frozen brain sections were obtained using a cryostat. Briefly, coronal sections (200 μm thick) were prepared, and the PVN was anatomically identified according to the rat brain atlas and carefully microdissected using a 1-mL syringe needle. Additional brain tissue containing the PVN was collected, fixed in 4% paraformaldehyde, and processed for paraffin embedding and subsequent immunohistochemical analysis. The harvested hearts were rapidly dissected, washed with cold normal saline to clear blood residue, and gently dried with blotting paper. Under ice-cold conditions, the great vessels and attached connective tissues were trimmed away, after which the heart weight was recorded. The heart weight index (HWI) was derived by normalizing heart weight to body weight (mg/g). Subsequently, the left ventricle was harvested and weighed for the calculation of left ventricular mass index (LVMI).

### 2.6 Western blotting (WB)

PVN samples were lysed in RIPA buffer (C1053, Applygen) and centrifuged at 12,000×g for 15 min at 4 °C. The protein content of each sample was quantified using a BCA assay kit (P1511, Applygen). Equal amounts of extracted proteins were separated by SDS-PAGE and electroblotted onto PVDF membranes. The membranes were blocked with 5% skim milk prepared in TBST for 2 h at room temperature, followed by incubation with specific primary antibodies diluted in blocking solution at 4 °C overnight. Primary antibodies against SIRT1 (1:500, DF6033, Affinity), NF-κB p65 (1:500, AF5006, Affinity), p-JNK (1:1000, AF3318, Affinity), p-p38 (1:1000, AF4001, Affinity), p-ERK1/2 (1:1000, AF1015, Affinity), β-actin (1:5000, 200068-8F10, Zenbio), and GAPDH (1:5000, 200306-7E4, Zenbio) were applied. HRP-linked goat anti-rabbit or anti-mouse secondary antibodies (1:5000, Zenbio) were used for incubation. Immunoreactive bands were detected with enhanced chemiluminescence reagent (34096, Thermo), and band intensity was analyzed by densitometry.

### 2.7 Immunohistochemistry

Paraffin-embedded brain tissue sections containing the PVN were placed in a 60 °C oven for 2 h, followed by deparaffinization in xylene and rehydration through a graded ethanol series. Antigen retrieval was performed by heating the sections in citrate buffer (pH 6.0) for 15 min. After cooling to room temperature, the sections were washed with PBS and incubated with 3% hydrogen peroxide to block endogenous peroxidase activity. Non-specific binding was blocked with 3% goat serum for 30 min at room temperature. The sections were then incubated overnight at 4 °C in a humidified chamber with primary antibodies against SIRT1 (1:100, DF6033, Affinity) and NF-κB p65 (1:150, AF6387, Affinity). After washing three times with PBS, the sections were incubated with the corresponding HRP-conjugated secondary antibody for 20 min at room temperature. Immunoreactivity was visualized using DAB, followed by counterstaining with hematoxylin. The sections were subsequently dehydrated through graded ethanol, cleared in xylene, and mounted with neutral resin. The stained sections were examined under a light microscope, and representative images were captured for evaluation of SIRT1- and NF-κB p65-positive cells in the PVN.

### 2.8 Quantitative real-time PCR (qRT-pCR)

The expression of angiotensin-converting enzyme (ACE), angiotensin II type 1 receptor (AT1R), tumor necrosis factor-α (TNF-α), interleukin-1β (IL-1β), interleukin-6 (IL-6), and monocyte chemoattractant protein-1 (MCP-1) in the PVN was quantified by qRT-PCR. The expression of atrial natriuretic peptide (ANP) and brain natriuretic peptide (BNP) in cardiac tissues was examined. Relative mRNA expression was calculated using the 2^–ΔΔCt^ method. Primer sequences are listed in Table 1.

**TABLE 1:** Primer sequences for qRT-PCR.

Gene	Forward	Reverse
ACE	CGTCCACCGTTACCAGACAA	TTGGCCTCTGCGTATTCGTT
AT1R	CAAAAGGAGATGGGAGGTCA	TGACAAGCAGTTTGGCTTTG
TNF-α	CGTCAGCCGATTTGCCATTT	TCCCTCAGGGGTGTCCTTAG
IL-1β	GGCTGACAGACCCCAAAAGA	TGTCGAGATGCTGCTGTGAG
IL-6	AGAGACTTCCAGCCAGTTGC	AGTCTCCTCTCCGGACTTGT
MCP-1	GTGCTGACCCCAATAAGGAA	TGAGGTGGTTGTGGAAAAGA
ANP	CCTGGACTGGGGAAGTCAAC	ATCTATCGGAGGGGTCCCAG
BNP	TTAGGTCTCAAGACAGCGCC	CGCCGATCCGGTCTATCTTC
GAPDH	TGACATCAAGAAGGTGGTGAAGC	GGAAGAATGGGAGTTGCTGTTG

### 2.9 Enzyme-linked immunosorbent assay (ELISA)

Plasma norepinephrine (NE), IL-6, IL-1β, PVN γ-aminobutyric acid (GABA), and PVN glutamate (Glu) levels were quantified using commercially available ELISA kits (Ke Zhong Zhi Biotechnology, Beijing, China). The following kits were used: NE (ZC-141697), IL-6 (ZC-141208), IL-1β (ZC-141195), GABA (ZC-141160), and Glu (ZC-141377). All assays were performed following the manufacturers’ recommended procedures.

### 2.10 Histological evaluation

Cardiac specimens fixed with paraformaldehyde were dehydrated, embedded in paraffin, and sectioned at 5 μm thickness. The tissue sections were placed onto glass slides, dried at 60 °C for 4 h, and stained with H&E and Masson staining according to established procedures. Histological alterations were evaluated using a light microscope, and images were captured at 400× magnification.

### 2.11 Statistical analyses

Data processing and statistical testing were completed using SPSS 25.0, with figures generated in Prism 9.5.0 (GraphPad). Data are expressed as mean ± SD. BP was analyzed using repeated-measures ANOVA, whereas other variables were analyzed using one-way ANOVA followed by the LSD test. *p* < 0.05 was considered statistically significant.

## 3 Results

### 3.1 Acupuncture attenuates BP in SHRs

To evaluate the antihypertensive effects of acupuncture, SBP and DBP were measured periodically during the experiment (Figures 2A,B). At all time points, the Model group exhibited significantly higher SBP and DBP than the Control group (*p* < 0.01). Compared with the Model group, SBP and DBP were notably reduced in the Losartan group from week 2 onward (*p* < 0.05), whereas significant reductions in the Acupuncture group were first observed at week 4 (*p* < 0.05). From week 6 onward, the Acupuncture and Losartan groups exhibited markedly lower SBP and DBP than the Model group, indicating a time-dependent antihypertensive effect of both interventions (*p* < 0.01). Further comparison between the two therapeutic groups revealed that DBP was lower in Losartan-treated rats (*p* < 0.01) than in Acupuncture-treated rats from week 8 onward, while a significant difference in SBP became apparent at week 10 (*p* < 0.05). At week 12, the Losartan group exhibited lower SBP and DBP (*p* < 0.01) than the Acupuncture group. These findings indicate that both acupuncture and losartan exerted significant antihypertensive effects in SHRs, while losartan produced an earlier and more pronounced BP-lowering effect. In this study, the Losartan group was included as a positive control to provide a reference for evaluating the antihypertensive efficacy of acupuncture.

**FIGURE 2:**
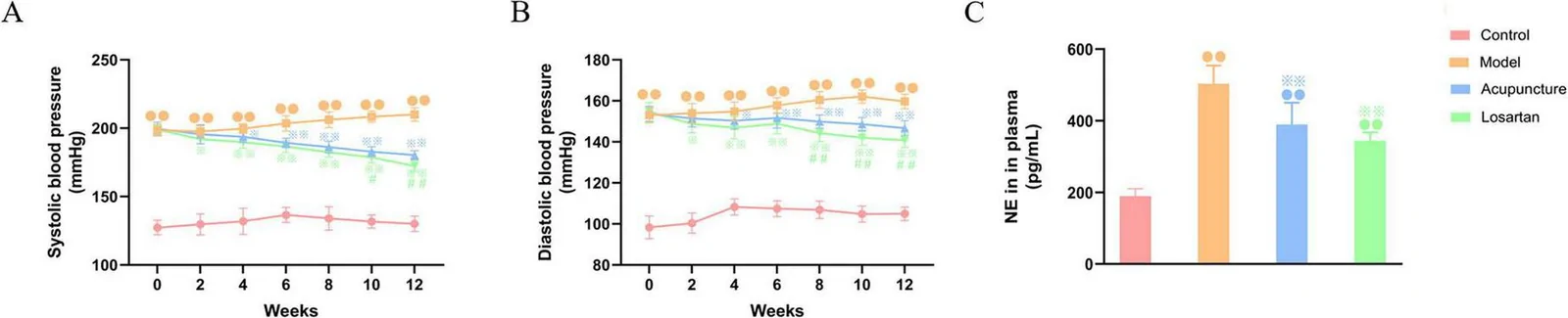
SBP **(A)**, DBP **(B)**, and NE **(C)** in SHRs after acupuncture treatment. ^••^*p* < 0.01, vs. Control group; ^⋇^*p* < 0.05, ^⋇⋇^*p* < 0.01, vs. Model group;^ #^*p* < 0.05, ^##^*p* < 0.01, vs. Acupuncture group.

### 3.2 Acupuncture attenuates sympathetic activation in SHRs

Plasma NE concentrations in the Control group were markedly lower (*p* < 0.01) than those in the Model group. In contrast, both the Acupuncture and Losartan groups exhibited significantly lower plasma NE concentrations (*p* < 0.01) relative to the Model group. These findings indicate that both acupuncture and losartan were associated with reduced plasma NE concentrations, which may reflect attenuation of sympathetic overactivity (Figure 2C).

### 3.3 Acupuncture modulates the SIRT1/NF-κB/MAPK signaling axis

WB analysis revealed that SIRT1 protein levels in the PVN were markedly lower in the Model group than in the Control group (*p* < 0.01) (Figures 3A,B). Meanwhile, the Model group showed markedly increased expression of NF-κB p65, p-JNK, p-p38, and p-ERK1/2 in the PVN (*p* < 0.01) (Figures 3C–E). Following intervention, both acupuncture and losartan treatment effectively restored SIRT1 protein levels (*p* < 0.05 or *p* < 0.01) and attenuated NF-κB p65, p-JNK, p-p38, and p-ERK1/2 protein levels (*p* < 0.01). Moreover, SIRT1 protein levels were higher in Losartan-treated rats than in Acupuncture-treated rats (*p* < 0.05), and the decreases in NF-κB p65, p-JNK, p-p38, and p-ERK1/2 levels were more pronounced (*p* < 0.01). Thus, although both interventions modulated the SIRT1/NF-κB/MAPK axis in the same direction, the magnitude of these changes was somewhat greater in the Losartan group than in the Acupuncture group.

**FIGURE 3:**
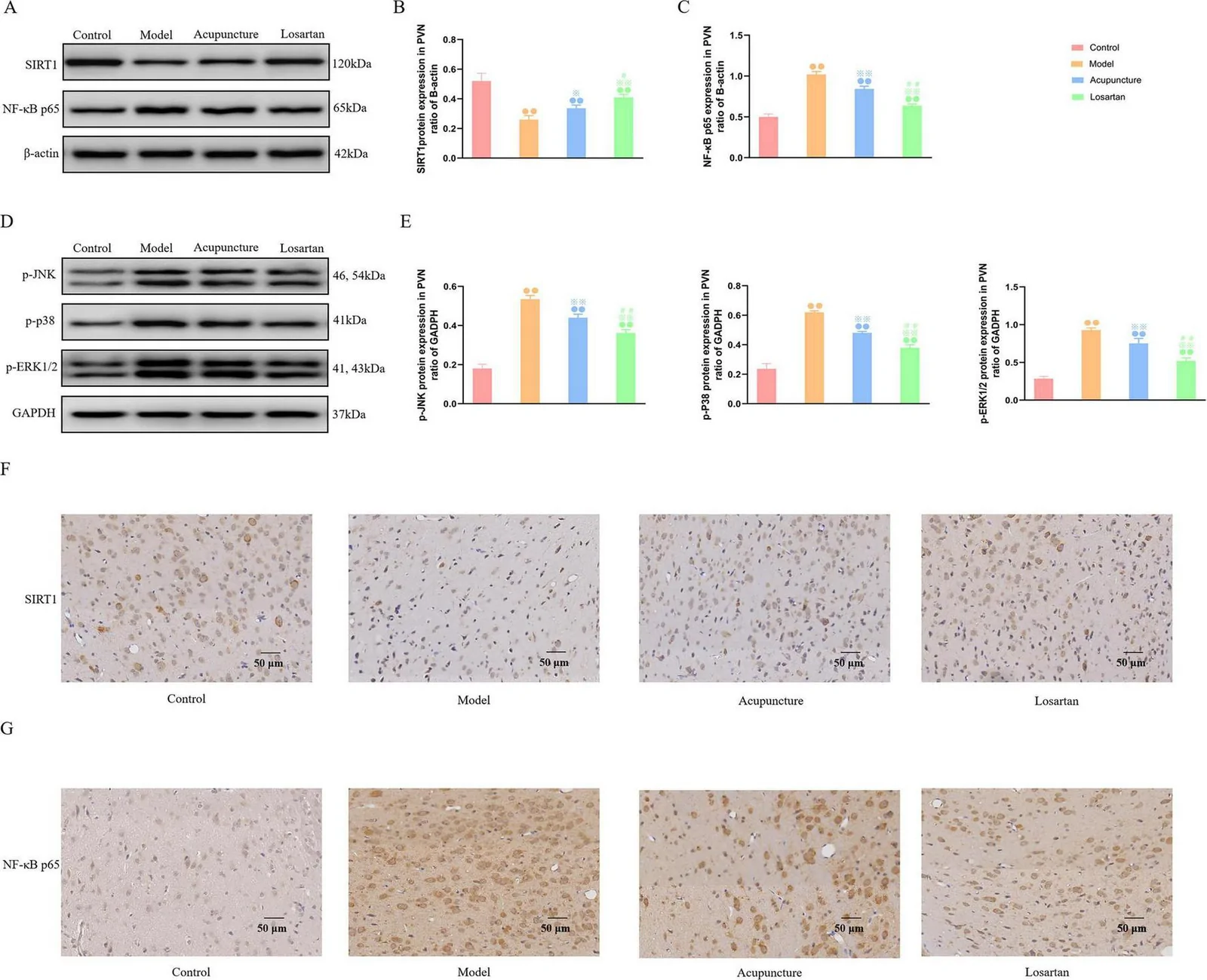
SIRT1/NF-κB/MAPK pathway-related protein expression within the PVN after acupuncture treatment. **(A)** Representative WB bands of SIRT1 and NF-κB p65 in the PVN. **(B)** SIRT1 protein expression in the PVN. **(C)** NF-κB p65 protein expression in the PVN. **(D)** Representative WB bands of MAPK pathway-related proteins in the PVN. **(E)** Relative protein expression levels of phosphorylated JNK, p38 MAPK, and ERK1/2 in the PVN. **(F)** SIRT1 immunohistochemical staining in the PVN. **(G)** NF-κB p65 immunohistochemical staining in the PVN. ^••^*p* < 0.01, vs. Control group; ^⋇^*p* < 0.05, ^⋇⋇^*p* < 0.01, vs. Model group; ^#^*p* < 0.05, ^##^*p* < 0.01, vs. Acupuncture group.

To further examine the expression of SIRT1 and NF-κB p65 in the PVN, immunohistochemical staining was performed. Representative images showed fewer SIRT1-positive cells and more NF-κB p65-positive cells in the Model group than in the Control group. Following acupuncture or losartan treatment, the number of SIRT1-positive cells appeared to increase, whereas the number of NF-κB p65-positive cells appeared to decrease compared with the Model group (Figures 3F,G). These observations were consistent with the changes detected by Western blotting and provided complementary histological evidence supporting a possible involvement of the SIRT1/NF-κB signaling pathway in the PVN.

### 3.4 Acupuncture modulates neurotransmitter levels in the PVN

The Model group exhibited markedly decreased GABA levels and increased Glu levels in the PVN (*p* < 0.01) relative to the Control group (Figures 4A,B). Following intervention, both acupuncture and losartan significantly enhanced GABA levels and reduced Glu levels in SHRs (*p* < 0.01). No significant differences were observed between the Acupuncture and Losartan groups in either GABA or Glu levels.

**FIGURE 4:**
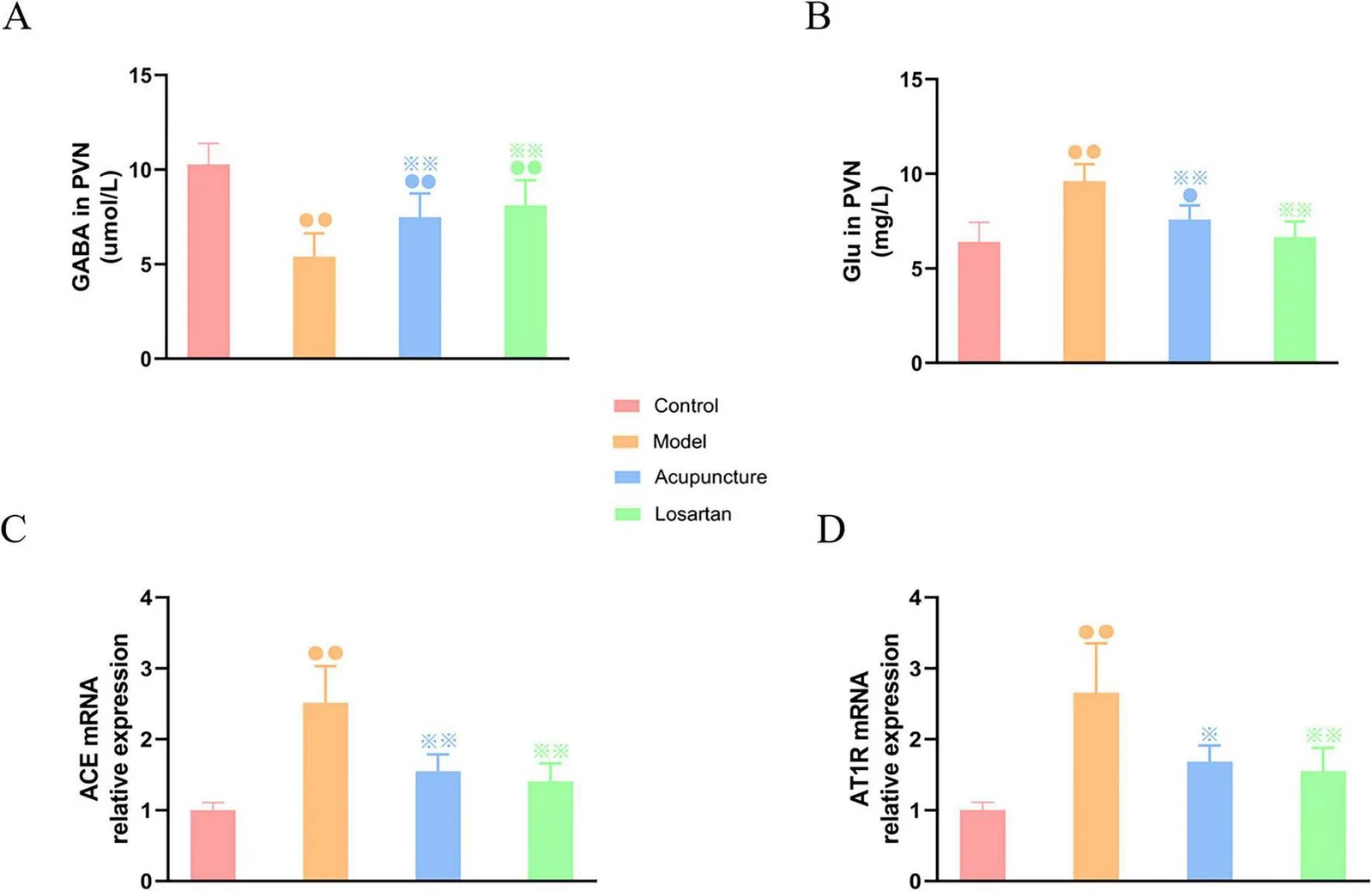
Neurotransmitter and key RAS component expression in the PVN after acupuncture treatment. **(A)** GABA levels. **(B)** Glu levels. **(C)** ACE mRNA expression. **(D)** AT1R mRNA expression. ^•^*p* < 0.05, ^••^*p* < 0.01, vs. Control group; ^⋇^*p* < 0.05, ^⋇⋇^*p* < 0.01, vs. Model group.

### 3.5 Acupuncture modulates key RAS components in the PVN

Relative to the Control group, ACE and AT1R mRNA expression levels in the PVN were significantly elevated in the Model group (*p* < 0.01) (Figures 4C,D). Following intervention, both acupuncture and losartan showed markedly reduced ACE and AT1R mRNA expression in SHRs (*p* < 0.05 or *p* < 0.01). No significant differences were observed between the Acupuncture and Losartan groups in PVN ACE or AT1R mRNA expression.

### 3.6 Acupuncture modulates pro-inflammatory signaling in serum and the PVN

Serum IL-6 and IL-1β levels in the Control group were markedly lower than those in the Model group (*p* < 0.01) (Figures 5A,B). Both acupuncture and losartan markedly decreased serum IL-6 and IL-1β levels in SHRs (*p* < 0.01). In the PVN, the Model group displayed higher levels of TNF-α, IL-1β, IL-6, and MCP-1 than the Control group (*p* < 0.01) (Figures 5C–F). Both acupuncture and losartan markedly downregulated the mRNA expression of these pro-inflammatory cytokines in SHRs (*p* < 0.01). No significant differences were observed between the Acupuncture and Losartan groups in serum or PVN pro-inflammatory markers.

**FIGURE 5:**
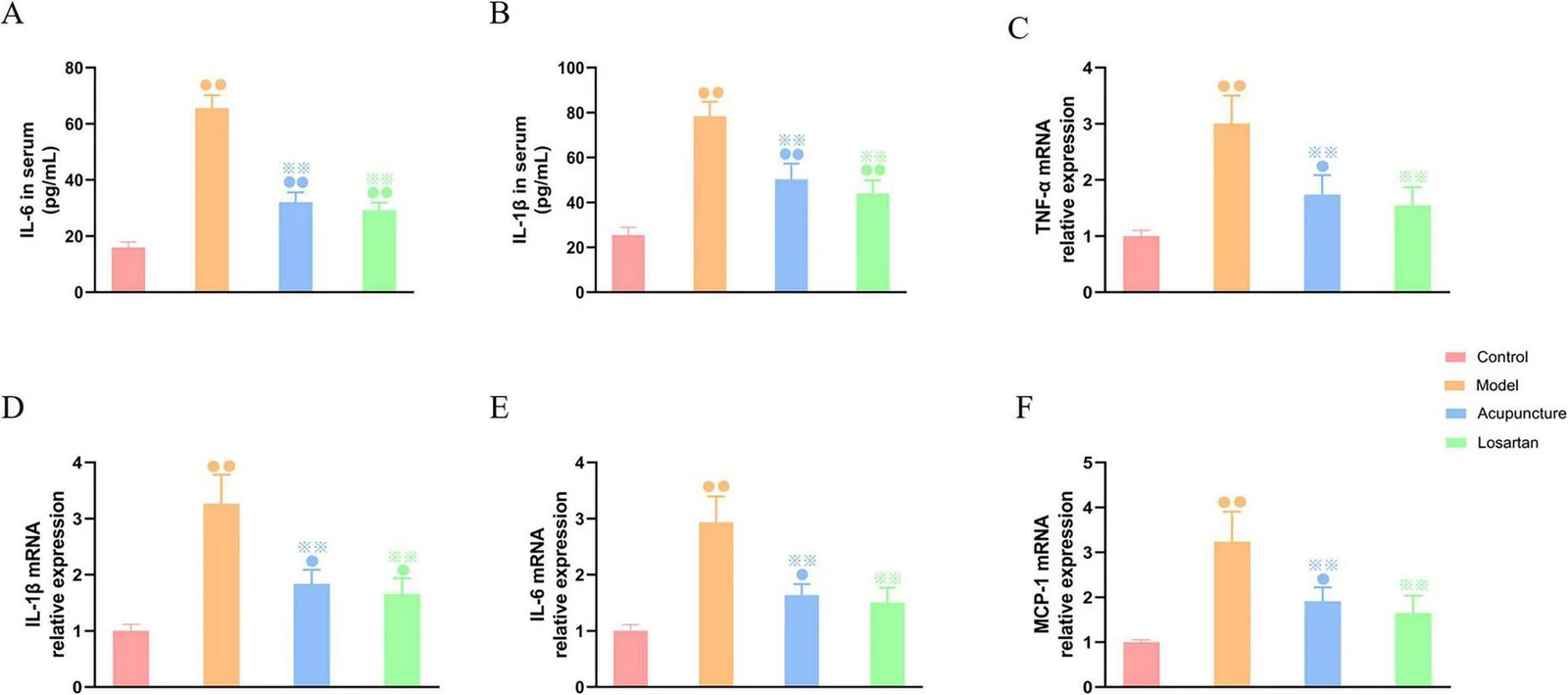
Pro-inflammatory cytokine expression in serum and the PVN following acupuncture treatment. **(A, B)** IL-6 and IL-1β in serum. **(C–F)** TNF-α, IL-1β, IL-6, and MCP-1 mRNA expression in the PVN. ^•^*p* < 0.05, ^••^*p* < 0.01, vs. Control group; ^⋇^*p* < 0.05, ^⋇⋇^*p* < 0.01, vs. Model group.

### 3.7 Acupuncture attenuates myocardial hypertrophy in SHRs

To evaluate myocardial hypertrophy, we measured the HWI (Figure 6A) and LVMI (Figure 6B). HWI and LVMI were both markedly lower in the Control group than in the Model group (*p* < 0.01). Following intervention, both acupuncture and losartan significantly reduced HWI and LVMI in SHRs (*p* < 0.01). Furthermore, Losartan-treated rats showed a greater reduction in LVMI than Acupuncture-treated rats (*p* < 0.05). To further explore the anti-hypertrophic mechanisms, the expression of myocardial hypertrophy markers ANP and BNP was measured by qRT-PCR. The Model group displayed a marked upregulation of ANP and BNP mRNA levels relative to the Control group (*p* < 0.01) (Figures 6C,D). Acupuncture and losartan both significantly reduced ANP and BNP mRNA expression in SHRs (*p* < 0.01). Overall, the two treatments were largely comparable in attenuating myocardial hypertrophy, with losartan showing a modestly greater reduction in LVMI.

**FIGURE 6:**
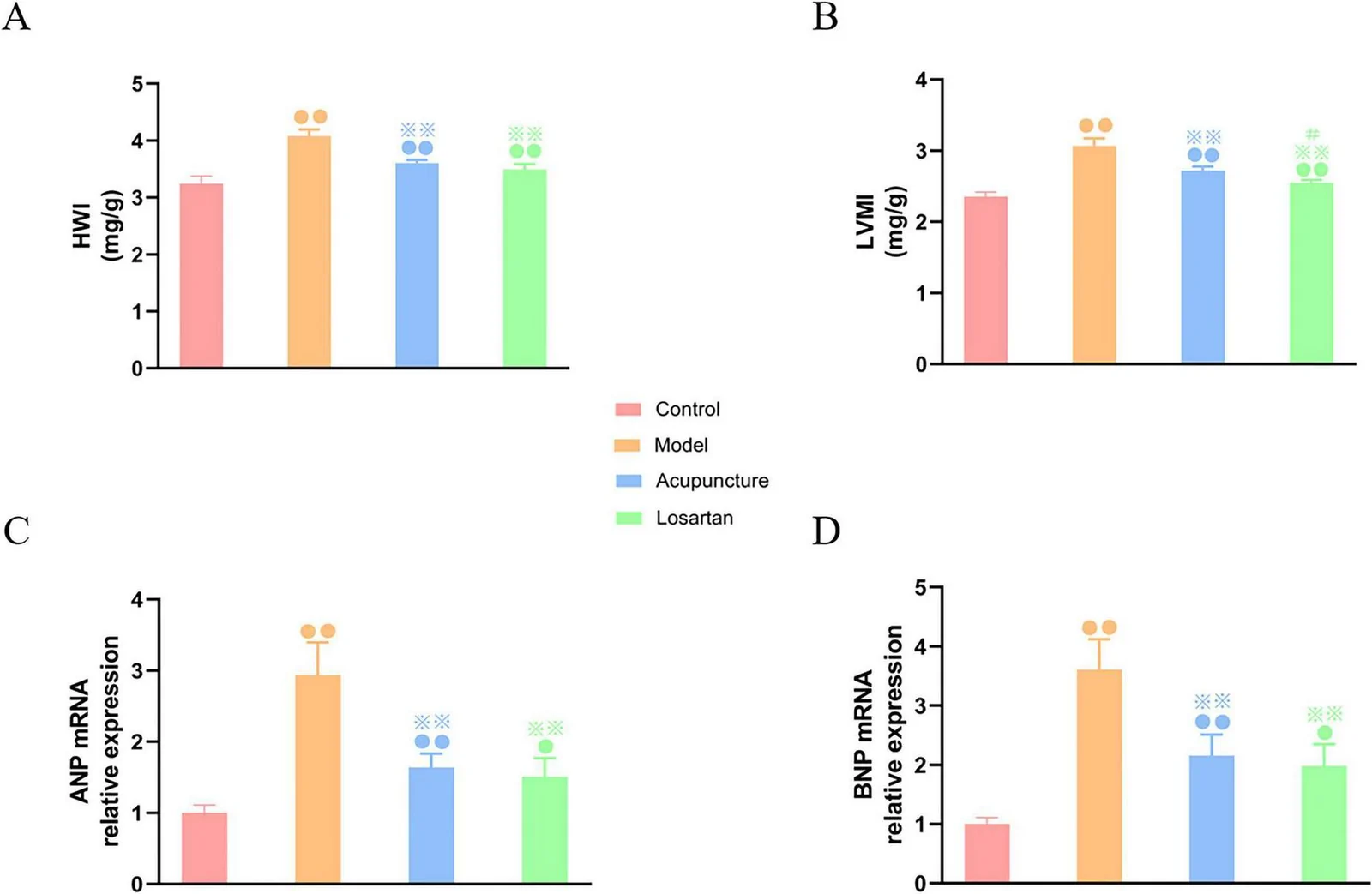
Alterations in myocardial hypertrophy-related parameters following acupuncture treatment. **(A)** HWI. **(B)** LVMI. **(C)** ANP mRNA expression. **(D)** BNP mRNA expression. ^•^*p* < 0.05, ^••^*p* < 0.01, vs. Control group; ^⋇⋇^*p* < 0.01, vs. Model group; ^#^*p* < 0.05, vs. Acupuncture group.

### 3.8 Acupuncture improves myocardial histological changes assessed by H&E staining

Myocardial fibers appeared disorganized, with increased intercellular spaces and apparent cardiomyocyte enlargement in the Model group. Altered nuclear morphology and increased inflammatory cell infiltration in the myocardial interstitium were also observed. In contrast, the Acupuncture and Losartan groups showed improved myocardial architecture, alleviated cardiomyocyte enlargement, and reduced inflammatory infiltration (Figure 7A).

**FIGURE 7:**
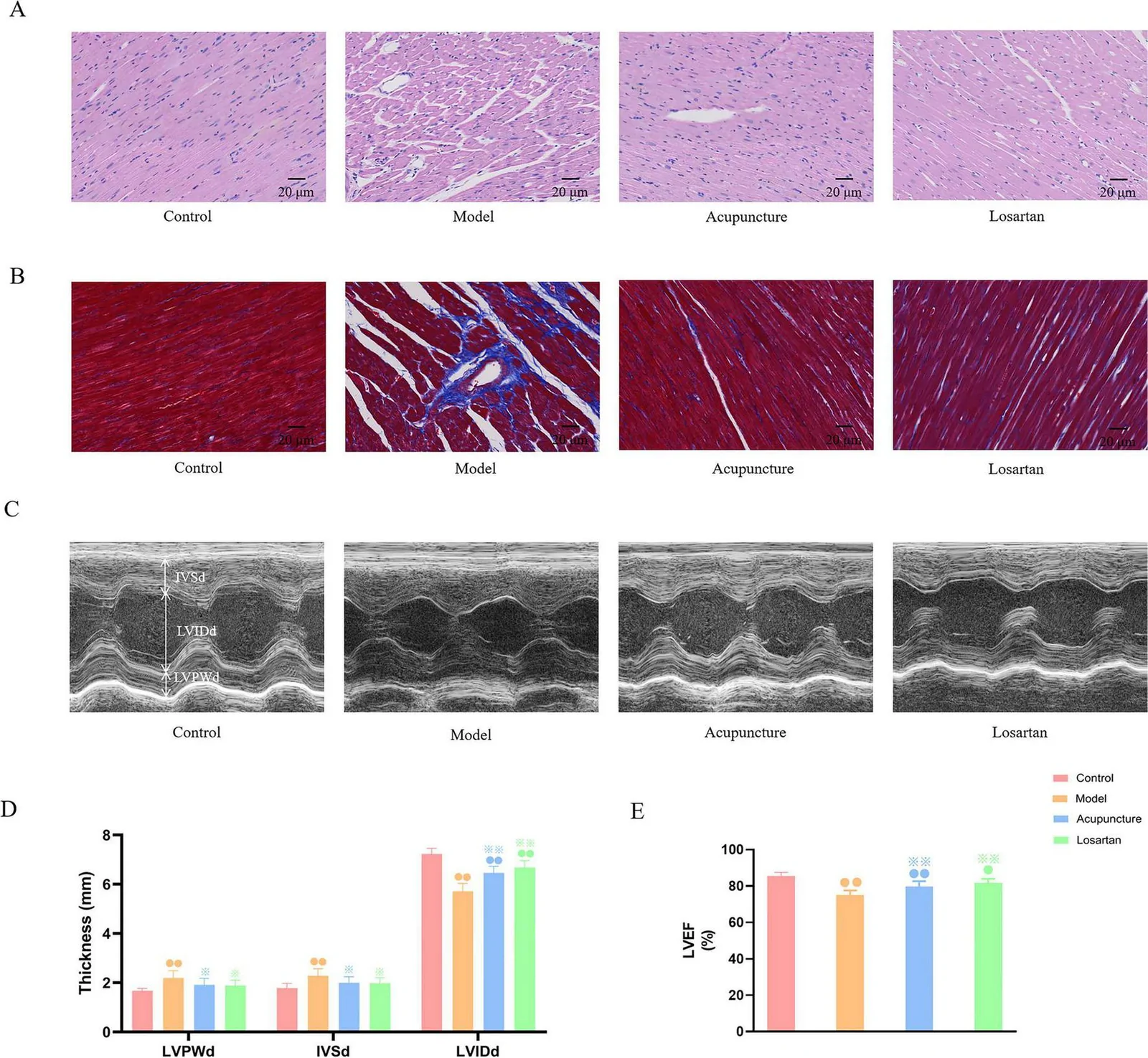
Myocardial histopathology and cardiac remodeling after acupuncture intervention. **(A)** Representative H&E-stained images demonstrating myocardial histopathological alterations. **(B)** Representative Masson’s trichrome-stained images illustrating myocardial collagen accumulation and interstitial fibrosis. **(C)** Representative M-mode echocardiographic images of rats from each group after completion of the 12-week intervention. **(D,E)** Echocardiographic parameters including LVPWd, IVSd, LVIDd, and LVEF. ^•^*p* < 0.05, ^••^*p* < 0.01, vs. Control group; ^⋇^*p* < 0.05, ^⋇⋇^*p* < 0.01, vs. Model group.

### 3.9 Acupuncture alleviates myocardial fibrosis in SHRs

Myocardial fibers in the Control group were tightly and orderly organized, and only limited blue-stained collagen fibers were observed around blood vessels. Myocardial fibers were disorganized and partially disrupted in the Model group, with increased cardiomyocyte cross-sectional width and marked interstitial accumulation of collagen fibers, reflecting myocardial hypertrophy accompanied by interstitial fibrosis. The Acupuncture and Losartan groups, relative to the Model group, displayed improved myocardial architecture, more orderly fiber arrangement, and a smaller blue-stained collagen area (Figure 7B).

### 3.10 Acupuncture improves cardiac remodeling in SHRs

After 12 weeks of intervention, M-mode echocardiographic images of the hearts were acquired from rats in each group, and representative images are shown in Figure 7C. Rats in the Model group showed markedly increased LVPWd and IVSd together with significantly lower LVIDd and LVEF than Control rats (all *p* < 0.01) (Figures 7D, E). Following intervention, both acupuncture and losartan significantly reduced LVPWd and IVSd in SHRs (*p* < 0.05), accompanied by elevated LVIDd and LVEF levels (*p* < 0.01), with no significant differences observed between the Acupuncture and Losartan groups for these echocardiographic parameters.

## 4 Discussion

Several limitations of the present study should be considered when interpreting our findings. First, although acupuncture upregulated SIRT1 and suppressed the NF-κB/MAPK axis in the PVN, the causal contribution of SIRT1 remains to be established; future studies combining PVN-targeted SIRT1 manipulation (e.g., knockdown or overexpression) with appropriate pharmacological modulation are needed to further clarify its mechanistic role. Second, although our findings highlight the PVN, hypertension involves multiple interconnected central autonomic networks, and the roles of other brain regions warrant further exploration. Third, BP was measured using a noninvasive tail-cuff system rather than radiotelemetry; despite standardized procedures, tail-cuff measurements cannot fully eliminate handling-related influences or provide continuous beat-to-beat recordings, and future telemetry-based studies are warranted to provide a more comprehensive assessment of BP dynamics. Fourth, the lack of a non-acupoint control group limits our ability to distinguish acupoint-specific effects from nonspecific effects of needling and handling; future studies with an appropriately matched control group are needed to further clarify the specificity of these effects. These limitations should be considered when interpreting the mechanistic associations identified in the present study.

The findings of our study suggest that acupuncture was associated with the amelioration of hypertension-induced myocardial hypaertrophy in SHRs. Acupuncture stimulation at LR3, GV20, and SP6 effectively reduced BP, inhibited sympathetic overactivity, and ameliorated cardiac remodeling. The underlying cardioprotective effects may involve restoration of SIRT1 expression and inhibition of the NF-κB/MAPK signaling pathways within the PVN, which may be associated with changes in the RAS system, pro-inflammatory cytokine expression, and excitatory–inhibitory neurotransmitter balance. These coordinated changes may contribute to the attenuation of sympathetic outflow and the subsequent improvement of cardiovascular function. Collectively, aour results indicate that modulation of the PVN SIRT1/NF-κB/MAPK signaling axis may be involved in the protective effects of acupuncture against hypertension-induced myocardial hypertrophy.

Hypertension is a major determinant of cardiovascular morbidity, and effective BP control is essential for reducing hypertension-related target organ damage associated with chronic hypertension (Jones and Clark, 2020; Unger et al., 2020). In this work, both acupuncture and losartan significantly reduced SBP and DBP in SHRs; however, their antihypertensive profiles differed. Losartan produced a marked reduction in BP as early as the second week of treatment, whereas the antihypertensive effect of acupuncture became statistically significant after four weeks and gradually accumulated throughout the intervention period. By the sixth week, both treatments maintained stable BP reduction. Thus, relative to acupuncture, losartan produced a faster onset and a somewhat greater magnitude of BP reduction. These findings further suggest that acupuncture may represent a valuable complementary strategy for BP management.

Excessive sympathetic activation is implicated in hypertension development (Grassi et al., 2023), and the PVN acts as an essential central hub for sympathetic regulation. Previous studies have demonstrated that an imbalance between Glu excitation and GABAergic inhibition within the PVN contributes to enhanced sympathetic activity (Grzęda et al., 2023; Zahner et al., 2024). Consistent with these findings, SHRs exhibited elevated plasma NE levels accompanied by increased Glu and decreased GABA levels within the PVN. Both acupuncture and losartan markedly reduced plasma NE levels and partially restored the PVN GABA/Glu balance. Of note, the reductions in plasma NE and the restoration of the GABA/Glu balance were comparable between the Acupuncture and Losartan groups. These findings support the hypothesis that acupuncture may modulate BP in association with the restoration of PVN neurotransmitter equilibrium and the alleviation of sympathetic overactivation (Jiao et al., 2025). Therefore, re-establishing neurotransmitter homeostasis within the PVN may represent a potential mechanism through which acupuncture may attenuate sympathetic overactivation and confer cardiovascular protection.

SIRT1, a NAD^+^-dependent deacetylase, negatively regulates NF-κB activation by facilitating p65 deacetylation, thereby suppressing the expression of pro-inflammatory cytokines (Yang et al., 2012; He et al., 2023). Meanwhile, the MAPK signaling pathway functions as an important modulator of inflammatory signal transduction, stress responses, and cellular dysfunction, and chronic activation of these pathways contributes to the progression of hypertension-associated neuroinflammation (Canovas and Nebreda, 2021). In this study, SHRs exhibited downregulated SIRT1 expression in the PVN, accompanied by upregulation of the NF-κB/MAPK signaling pathways, which may contribute to increased pro-inflammatory cytokine expression. These findings suggest that acupuncture may exert central anti-inflammatory effects in the PVN, a process that is accompanied by modulation of the SIRT1/NF-κB/MAPK signaling axis, with concurrent reductions in neuroinflammatory responses and alleviation of sympathetic overactivity, which may jointly contribute to the improvement of hypertension-induced cardiac remodeling. The RAS is a principal regulator of cardiovascular homeostasis, and its aberrant activation has been implicated in the development and progression of hypertension (Su et al., 2021; Kanugula et al., 2023). Beyond the circulating RAS, a locally regulated RAS system exists within the brain (de Morais et al., 2018; Urmila et al., 2021). In our study, SHRs exhibited a significant upregulation of ACE and AT1R expression within the PVN, whereas both acupuncture and losartan were associated with marked reductions in the expression of these key RAS components. Comparison of the two intervention groups further revealed that the modulation of the PVN SIRT1/NF-κB/MAPK signaling axis appeared more pronounced in the losartan group, whereas the reductions in PVN ACE and AT1R expression were comparable between the Acupuncture and Losartan groups. Taken together, these findings suggest that acupuncture and losartan may share certain effects on PVN RAS-related signaling, while their effects on the SIRT1/NF-κB/MAPK signaling axis may differ in magnitude. Given the established interaction between central RAS signaling, inflammatory pathways, and sympathetic regulation, these findings raise the possibility that modulation of local RAS signaling within the PVN may contribute to the cardiovascular effects of acupuncture. However, this interpretation remains associative and does not establish that acupuncture directly restores central RAS homeostasis or that RAS modulation mediates the effects of acupuncture. Consistent with the present findings, accumulating evidence indicates that the PVN is a key site at which local RAS signaling, particularly Ang II/AT1R activation, is involved in sympathetic overactivity and BP elevation (Mińczuk et al., 2022). In addition, recent studies have shown that acupuncture can modulate PVN Ang II/AT1R signaling in SHRs, with concomitant reductions in sympathetic outflow and BP (Jiao et al., 2025), which provides a plausible mechanistic context for the present findings. Nevertheless, because the present study did not include PVN-targeted pharmacological or genetic manipulation, these findings support an association between PVN RAS modulation and the cardiovascular effects of acupuncture but do not establish a direct causal relationship.

Chronic hypertension promotes progressive pathological remodeling of the heart, characterized by myocardial hypertrophy, interstitial fibrosis, and impaired cardiac function (Nwabuo and Vasan, 2020; Masenga and Kirabo, 2023). In our experiments, SHRs exhibited significantly increased HWI and LVMI, accompanied by upregulated ANP and BNP mRNA levels. Histological analyses using H&E and Masson staining further demonstrated disorganized myocardial fibers, enlarged cardiomyocyte cross-sectional area, infiltration of inflammatory cells, and excessive collagen accumulation within the myocardium. Echocardiographic assessment revealed increased LVPWd and IVSd, together with decreased LVIDd and LVEF, indicating marked left ventricular remodeling and cardiac dysfunction. Both acupuncture and losartan effectively ameliorated hypertension-induced myocardial hypertrophy, as demonstrated by decreased cardiac hypertrophy indices, reduced expression of hypertrophic markers ANP and BNP, attenuation of myocardial fibrosis, and restoration of cardiac function. Although losartan showed a greater improvement in some parameters, acupuncture also produced marked antihypertrophic effects and mitigated cardiac remodeling, suggesting its promise as a complementary treatment strategy for hypertension-induced myocardial hypertrophy.

## 5 Conclusion

In conclusion, acupuncture was associated with the attenuation of hypertension-induced myocardial hypertrophy in SHRs. These beneficial effects may be associated with modulation of the SIRT1/NF-κB/MAPK signaling axis in the PVN, along with changes in the PVN renin–angiotensin system, pro-inflammatory cytokine expression, and neurotransmitter balance. Collectively, these findings provide new insights into the potential central mechanisms underlying acupuncture-mediated cardioprotection in hypertension and suggest that acupuncture may serve as a complementary therapeutic approach for attenuating hypertension-related target organ damage.

## Data Availability

The original contributions presented in the study are included in the article/supplementary material, further inquiries can be directed to the corresponding author.
